# Efficient role of IgH 3′ regulatory region deficient B-cells in the development of oil granulomas

**DOI:** 10.18632/oncotarget.9588

**Published:** 2016-05-25

**Authors:** Nour Ghazzaui, Alexis Saintamand, Hussein Issaoui, Faten Saad, Yves Denizot

**Affiliations:** ^1^ Department of Immunology, CNRS UMR 7276, CRIBL, Université de Limoges, Limoges, France

**Keywords:** IgH 3′ regulatory region, knock-out mice, pristine, granulomas

## Abstract

Functional B-cells are essential for the formation of oil granulomas. The IgH 3′ regulatory region (3′RR) activates important check-points during B-cell maturation. We investigated if 3′RR-deficient B-cells remain efficient to develop oil granulomas in response to pristine. B-cells expressing an IgH 3′RR-deficient allele were similarly recruited to wild type allele expressing B-cells in the granuloma. No differences were observed between 3′RR-deficient mice and control mice for granuloma numbers, cellular composition and ability to express mRNA transcripts for several pro- and anti-inflammatory cytokines. Altogether these results suggest a normal role for 3′RR-deficient B-cells in the development of an acute B-cell-mediated inflammatory response.

## INTRODUCTION

The IgH locus undergoes multiple changes along B-cell differentiation, affecting transcription and accessibility to V(D)J recombination, somatic hypermutation (SHM) and class switch recombination (CSR) [1, 2]. IgH *cis*-regulatory regions are major locus regulators. The IgH 3′ regulatory region (3′RR) promotes SHM [3, 4], CSR [5–7], μ transcription [8] but not V(D)J recombination [9]. The 3′RR is also a potent lymphoma oncogene deregulator [10]. Transgenic mice models demonstrated the 3′RR implication in the development of several B-cell lymphomas [11–18]. The absence of the 3′RR influences lymphomagenesis in λ-myc mice toward less mature lymphomas [19]. Until now, the functionality of 3′RR-deficient B-cells in inflammatory response is poorly documented. The lack of 3′RR was only reported to marginally impact the development of a chronic inflammatory ascite formation in response to pristine [20].

The *i.p.* injection of pristine induces the formation of mesenteric oil granulomas [21, 22]. Pristine-induced granulomas are characterised by clustered cells adhered to the mesentery and other peritoneal tissues. The granuloma formation constitutes a protective inflammatory cellular response of the host against invading pathogens or indigestible substances. Two types of granulomas are reported. Serosal granulomas (SG) are located at the interface of the mesenteric margins and gut. Mesenteric granulomas (MG) are located around the center of the mesenteric tissue [23, 24]. Oil granulomas are considered as tertiary lymphoid tissues constituted of monocytes, granulocytes, T-cells and B-cells. Their formation is regulated by several cytokines [25]. The absence of functional B-cells blocks SG formation and diminishes MG development in response to pristine [23]. Mesenteric oil granulomas thus appear as an interesting tool to ensure the functional ability of 3′RR-deficient B-cells in the occurrence and/or development of an acute inflammatory response. In this study we investigated the generation of pristine-induced oil granulomas in IgH 3′RR-deficient mice.

## RESULTS

### Spleen and peritoneal B-cells expressing a 3′RR-deleted allele

**Figure 1:**
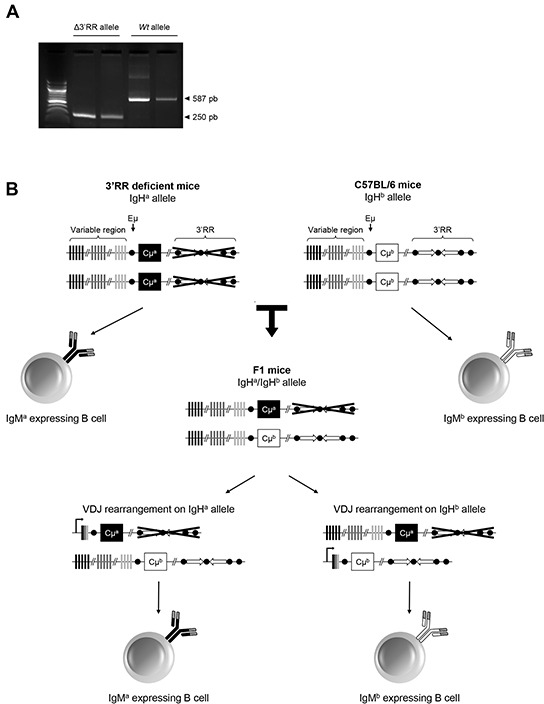
Generation of a^Δ3′RR^/b*^wt^* and a*^wt^*/b*^wt^* mice **A.** PCR profile for a 3′RR-deficient and a *wt* IgH allele. **B.** Backcross for generation of a^Δ3′RR^/b*^wt^* and a*^wt^*/b*^wt^* mice, and expression of either IgM^a^ or IgM^b^ allele by B-cells from F1 mice. B-cell are expected to express IgM^a^ or IgM^b^ at similar frequency, including when the *a* allele is deleted for the 3′RR, since its deletion does not affect VDJ recombination. If the expression of one of this allele impedes the B-cell development, the equilibrium between IgM^a^ or IgM^b^ expressing B-cell will be disrupted. Lowered number of IgM^a^ expressing B-cells in a^Δ3′RR^/b*^wt^* mice will thus demonstrate that deletion of the 3′RR alters B-cell development or recruitment.

**Figure 2:**
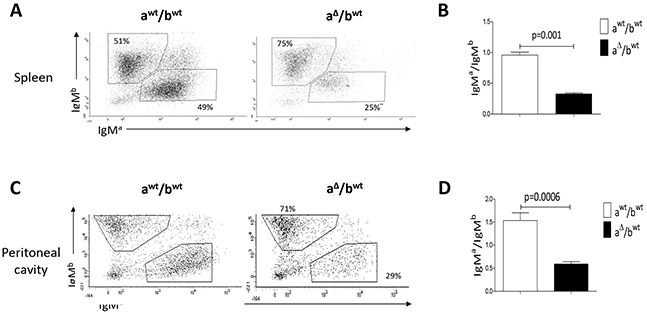
IgM^a^ and IgM^b^ in spleen and peritoneal cavity of heterozygous IgH a^Δ3′RR^/b*^wt^* and a*^wt^*/b*^wt^* mice **A.** Flow cytometry analysis of the percentages of IgM^a^ and IgM^b^ in spleen of a^Δ3′RR^/b*^wt^* and a^wt^/b*^wt^* mice. Cells were gated on B220^+^ cells. One representative experiment out of six a^Δ3′RR^/b*^wt^* mice and nine a^wt^/b*^wt^* mice is shown. **B.** IgM^a^/IgM^b^ ratio in splenocytes of a^Δ3′RR^/b*^wt^* and a^wt^/b*^wt^* mice. Mean ± SEM of six a^Δ3′RR^/b*^wt^* mice and nine a^wt^/b*^wt^* mice. Significance was assessed using the Mann-Whitney *U*-test. **C.** Flow cytometry analysis of the percentages of IgM^a^ and IgM^b^ in the peritoneal cavity of a^Δ3′RR^/b*^wt^* and a^wt^/b*^wt^* mice. Cells were gated on B220^+^ cells. One representative experiment out of seven for both genotypes is shown. **D.** IgM^a^/IgM^b^ ratio in the peritoneal cavity of a^Δ3′RR^/b*^wt^* and a^wt^/b*^wt^* mice. Mean ± SEM of seven experiments. Significance was assessed using the Mann-Whitney *U*-test.

Mouse substrains have dissimilar differentiation programs culminating in different B-cell fate, BCR expression and signalling [8]. Pristine-induced oil granuloma generation is different with respect to mouse substrains [24]. Before assessing the influence of an IgH 3′RR-deleted allele *vs* a *wt* allele in B-cell recruitment in oil granulomas we firstly investigated heterozygous IgH a^Δ3′RR^/b*^wt^* mice. The presence of a 3′RR-deficient allele and a *wt* allele was investigated by PCR (Figure 1A). The 3′RR deletion was done in a 129 ES cell line (IgH *a* allotype) and developed in a 129 background (IgH a^*wt*^/a^*wt*^). Heterozygous IgH a^Δ3′RR^/b*^wt^* mice were generated by crossing homozygous 3′RR-deficient mice (IgH a^Δ3′RR^/a^Δ3′RR^) with C57BL/6 mice bearing an IgH *b* allotype (IgH b*^wt^*/b*^wt^*) mice (Figure 1B). Mixed 129 x C57BL/6 mice (IgH a*^wt^*/b*^wt^*) were used as control mice. As previously reported [8], analysis of splenic B-cells with IgM-allotype specific antibodies indicated a lowered (p=0.001, Mann-Whitney *U*-test) percentage of B-cells expressing an *a* allotype (IgM*^a^*/IgM*^b^* ratio: 0.33) in a^Δ3′RR^/b*^wt^* mice (Figure 2A and 2B). A similar decrease (p=0.0006) was also found for peritoneal B-cells (IgM*^a^*/IgM*^b^* ratio: 0.59) (Figure 2C and 2D). While similar percentages of B-splenocytes expressed either an *a* or *b* allotype (IgM*^a^*/IgM*^b^* ratio: 0.96) in a*^wt^*/b*^wt^* mice (Figure 2A and 2B), elevated number of peritoneal B-cells expressing an *a* allotype was found (IgM*^a^*/IgM*^b^* ratio: 1.53) (Figure 2C and 2D). This result might be linked to a differential strength of signalling between IgM^a^ BCR and IgM^b^ BCR for proliferation/survival of peritoneal B-cells. Such specific interactions with IgM^a^ (but not IgM^b^) determinants have been already reported with the HIV-1 envelope gp41 membrane proximal external region [26]. Furthermore, the phenotype of mature B-cells differs between the various mouse substrains. Notably, BCR signalling has been suggested to be lower in 129 mice than in C57BL/6 [27]. Altogether these results suggest that the 3′RR deletion is not only detrimental for efficient B-cell maturation in spleen but also for B-cells in the peritoneal cavity.

### B-cells expressing a 3′RR-deleted allele in oil granulomas

**Figure 3:**
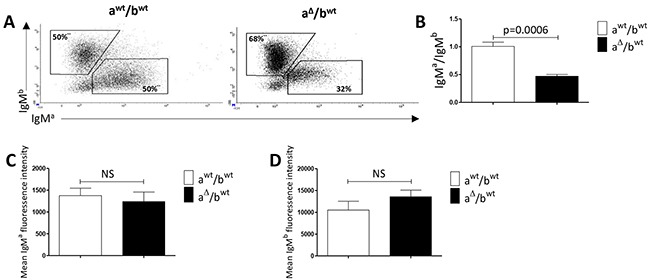
IgM^a^ and IgM^b^ in oil granulomas of heterozygous IgH a^Δ3′RR^/b*^wt^* and a*^wt^*/b*^wt^* mice **A.** Flow cytometry analysis of the percentages of IgM^a^ and IgM^b^ in granulomas of a^Δ3′RR^/b*^wt^* and a^wt^/b*^wt^* mice. Cells were gated on B220^+^ cells. One representative experiment out of eight a^Δ3′RR^/b*^wt^* mice and nine a^wt^/b*^wt^* mice is shown. **B.** IgM^a^/IgM^b^ ratio in granulomas of a^Δ3′RR^/b*^wt^* and a^wt^/b*^wt^* mice. Mean ± SEM of eight a^Δ3′RR^/b*^wt^* mice and nine a^wt^/b*^wt^* mice. Significance was assessed using the Mann-Whitney *U*-test. **C.** Mean ± SEM membrane IgM^a^ density in granulomas of seven a^Δ3′RR^/b*^wt^* mice and eight a^wt^/b*^wt^* mice. NS: not significant (Mann-Whitney *U*-test). **D.** Mean ± SEM membrane IgM^b^ density in granulomas of seven a^Δ3′RR^/b*^wt^* mice and eight a^wt^/b*^wt^* mice. NS: not significant (Mann-Whitney *U*-test).

We next compared B-cell recruitment in granulomas from a^Δ3′RR^/b*^wt^* and a*^wt^*/b*^wt^* mice. For granuloma experiments we used mechanical dissociation instead of collagenase dissociation. Collagenase-based intestinal digestion procedure is frequently used to isolate tissue-resident B-cells. However this procedure was recently reported to alter B-cell surface marker expression and thus can impede the correct phenotypic analysis of these B-cells [28]. All granulomas were investigated the same day to ensure similar recovery efficiency. As a positive control, similar percentages of B-cells expressing either a *a* or *b* allotype (IgM*^a^*/IgM*^b^* ratio: 1.00) were found in granulomas of a*^wt^*/b*^wt^* mice (Figure 3A and 3B). Analysis of B-cells in oil granulomas with IgM-allotype specific antibodies indicated a lowered (p=0.0006) percentage of B-cells expressing an *a* allotype (IgM*^a^*/IgM*^b^* ratio: 0.47) in a^Δ3′RR^/b*^wt^* mice (Figure 3A and 3B). The 53% of IgM^a^ reduction paralleled the 41% and 67% of IgM^a^ reduction in peritoneal cavity and spleen of IgH a^Δ3′RR^/b*^wt^* mice, respectively. Finally the mean membrane IgM^a^ and IgM^b^ densities were similar in heterozygous a^Δ3′RR^/b*^wt^* and a*^wt^*/b*^wt^* mice (Figure 3C and 3D). Thus, differences in IgM^a^ and IgM^b^ allotypes in oil granulomas in heterozygous a^Δ^/b*^wt^* mice are linked to differences in the percentage of IgM^a^ and IgM^b^ B-cells in mice but not to a defect in B-cell recruitment of 3′RR-deficient B-cells.

### Oil granulomas in 3′RR-deficient mice and *wt* mice

**Figure 4:**
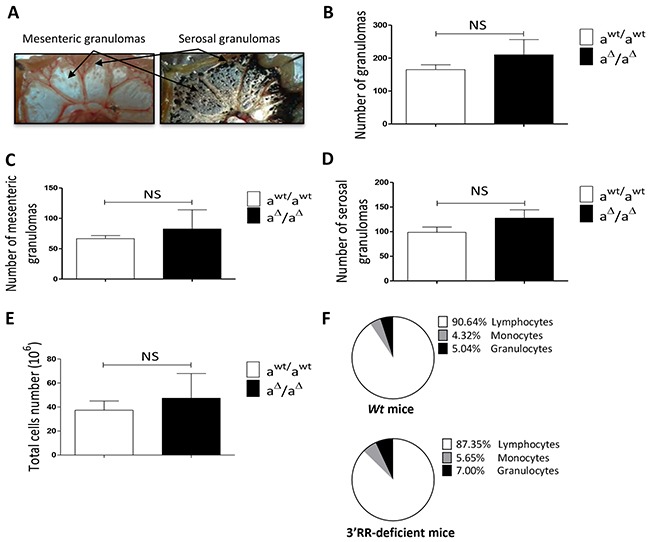
Oil granulomas in homozygous 3′RR-deficient mice **A.** Response to the mesentery to pristine in 3′RR-deficient mice. After *i.p.* injection of 1ml of pristine, mice were sacrificed at week 3. The gut associated whole mesenteric tissue was photographed (right part). In another set of experiment 2 week before sacrifice mice were injected i.p. with 0.5 ml of a 1/10 dilution of India ink in PBS. Since pristine droplets are surrounded by phagocytes, carbon particules were internalised into phagocytes better highlighting mesenteric and serosal granulomas. Black arrows locate mesenteric granulomas (MG) and serosal granulomas (SG). One representative experiment out of 10 is shown. **B-D.** Number of total granulomas (B), mesenteric granulomas (C) and serosal granulomas (D) in 3′RR-deficient and *wt* mice. Mean ± SEM of four 3′RR-deficient mice and six *wt* mice. NS: not significant (Mann-Whitney *U*-test). E: Total cell number in the gut associated whole mesenteric tissue. Mean ± SEM of four 3′RR-deficient mice and six *wt* mice. NS: not significant (Mann-Whitney *U*-test). F: Percentage of lymphocytes, monocytes and granulocytes in the gut associated whole mesenteric tissue. Mean ± SEM of four 3′RR-deficient mice and six *wt* mice.

We next compared granuloma formation in 3′RR-deficient mice (IgH locus a^Δ3′RR^/a^Δ3′RR^) and *wt* mice (IgH locus a*^wt^*/a*^wt^*). A representative photograph of the gut associated whole mesenteric tissue 3 weeks after *i.p.* injection of 1ml pristine is reported in Figure 4A. To contrast with the background we labelled phagocytes with India ink by injecting it intraperitoneally into mice at week 1 after pristine (Figure 4A). Arrows indicate locations of MG and SG. For all experiments we counted granulomas on the whole mesenteric tissue. Numbers of total granulomas (Figure 4B), MG (Figure 4C) and SG (Figure 4D) were not significantly different between 3′RR-deficient mice and *wt* mice. No significant differences were found for the total cell number in the gut associated whole mesenteric tissue (MG + SG) between 3′RR-deficient mice and *wt* mice (Figure 4E). The percentages of granulocytes, monocytes and lymphocytes (morphological analysis and counts in the CELL-DYN Emerald) were not significantly affected in the gut associated whole mesenteric tissue (MG + SG) of 3′RR-deficient mice (Figure 4F). Finally flow cytometry analysis indicated similar (p=1, Mann-Whitney *U*-test) percentages of IgM^a+^ B-cells in granulomas of 3′RR-deficient mice (32.6 ± 5.8 %, mean ± SEM of 3 animals) and *wt* mice (40.7 ± 13.2 %, mean ± SEM of 3 animals). Taken altogether these results suggest no impact of the 3′RR deficiency for oil granuloma formation.

### Inflammatory cytokine network in oil granuloma in 3′RR-deficient mice and *wt* mice

Cytokines have been reported to regulate the structure and formation of oil granulomas in mice [25, 29]. Several pro- (INF-γ, TNF-α, CXCL2, IL-12, IL-6) and anti-inflammatory (IL-4, IL-10, TGF-β) cytokine transcripts were investigated, by real-time PCR, in granulomas cells of 3′RR-deficient and *wt* mice. Adherent (monocytes/macrophages) and non-adherent (lymphocytes/granulocytes) cells were investigated to search putative differences between 3′RR-deficient and *wt* mice. As shown in Table 1, no significant differences were found for INF-γ, TNF-α, CXCL2, IL-12, IL-6, IL-4, IL-10, TGF-β mRNA transcripts between 3′RR-deficient mice and *wt* mice. These results reinforce the hypothesis of a similar mechanistic/kinetic of granuloma formation in mice with 3′RR-deficient B-cells and *wt* B-cells.

**Table 1:** INF-γ, TNF-α, CXCL2, IL-12, IL-6, IL-4, IL-10 and TGF-β mRNA transcripts in adherent and non-adherent granuloma cells from 3′RR-deficient mice and wt mice

	*Non-adherent cells*			*Adherent cells*		
*Cytokines*	*Wt* mice	3′RR-deficient mice	Significance	*Wt* mice	3′RR-deficient mice	Significance
***IL-10***	102.2 ± 24.0	170.7 ± 184.6	1.0	18.3 ± 18.2	57 ± 59.5	0.7
***IL-4***	22.3 ± 10.4	5.5 **±** 3.0	0.1	187.6 ± 92.6	58.1 ± 68.8	0.2
***TGF-β***	10.0 ± 5.7	7.3 ± 4.8	0.8	18182.8 ± 13113.5	17473.4 ± 12895.2	1.0
***IL-6***	104.6 ± 127.0	265.6 ± 451.4	1.0	12043.4 ± 15219.9	37439.7 ± 57409.8	1.0
***TNF-α***	21.4 ± 2.8	26.9 ± 32.9	0.4	432.6 ± 493.9	615.2 ± 585.8	0.6
***IL-12***	736.5 ± 244.3	1448.1 ± 1346.8	0.6	1044.1 ± 1111.5	2038.1 ± 2411.3	1.0
***INF-γ***	41.0 ± 12.8	50.7 ± 34.0	0.6	64 ± 13.4	60.06 ± 72.6	1.0
***CXCL2***	31.1 ± 15.3	43.3 ± 64.6	0.4	612.3 ± 704.4	543.3 ± 612.4	1.0

Gene expression levels were normalized to GAPDH mRNA. Amounts of transcripts were compared to sample with the lowest level of transcripts. Results are reported as Mean ± SEM from 4 independent experiments per group. Significance was assessed with the Mann-Whitney U-test. No significant differences were documented between groups.

## DISCUSSION

The IgH 3′ regulatory region (3′RR) stimulates numerous important B-cell check-points during B-cell maturation [3–8]. We have investigated the impact of the 3′RR deletion on the *in vivo* pristine-induced granuloma formation. By using heterozygous a^Δ3′RR^/b^*wt*^ mice we demonstrated that B-cells expressing a 3′RR-deficient allele are efficiently recruited in the granuloma structure. The 3′RR controls μ chain expression and 3′RR-deficient B-cells expressed reduced levels of membrane BCR [8]. BCR signalling is not only essential for normal B-cell development but also for B-cell-mediated inflammation via cytokine production and regulation of T-cell response [30]. The reduced BCR expression at the membrane of 3′RR-deficient B-cells is, thus, not crippling to generate an appropriated inflammatory response in the oil granuloma model. By using homozygous 3′RR-deficient mice we demonstrated a similar granuloma response compared to *wt* mice. Oil granuloma formation was severely abrogated in B-cell-deficient mice while T-cells were dispensable for pristine-induced oil granuloma formation [23]. Functional B-cells are thus required for the initiation and development of oil granulomas. Our results with 3′RR-deficient mice indicate that a fully efficient CSR, SHM, BCR expression and μ transcription are not mandatory for the initiation/development of oil granulomas. Inflammation induces local expression of chemokines that attract leukocytes into the site of inflammation. The local balance between pro-inflammatory and anti-inflammatory cytokines is also of importance for the initiation/development of oil granulomas [23, 25, 31]. Pristine activates resident peritoneal cells such as B-cells and monocytes/macrophage leading to the secretion of various cytokines. Analysis of several pro-inflammatory (INF-γ, TNF-α, CXCL2, IL-12, IL-6) and anti-inflammatory (IL-4, IL-10, TGF-β) cytokines by granuloma cells did not evidenced any differences between 3′RR-deficient mice and *wt* mice. These results reinforce the hypothesis of a similar mechanistic/kinetic of granuloma formation in mice with 3′RR-deficient B-cells and *wt* B-cells.

In conclusion the 3′RR targeting has no significant effect on the acute inflammatory B-cell-mediated oil granuloma model. The 3′RR is a major lymphoma oncogene deregulator [10–19]. The 3′RR might be considered as a potential target for anti-lymphoma pharmacological therapy without significant impact on the normal immune and inflammatory networks [32]. 3′RR activation and transcriptional activity are altered by a diverse range of chemicals, including ones with anti-carcinogenic properties [33]. Histone deacetylase inhibitors (HDACi) might be of interest since 3′RR-induced activation is mediated through activation of specific epigenetic marks [7] and since the hs1,2 enhancer (located in the central palindromic 3′RR structure) is sensitive to HDACi [34]. A limitation of the pristine mouse model is that inflammation is restricted to the peritoneal cavity. Other mice models of inflammatory reactions must be tested before definitive validation of this hypothesis such as the pathogenic role of B-cells in the development of diffuse alveolar hemorrhage induced by pristine [35] and the inflammatory pathology induced by surgical implants [36].

## MATERIALS AND METHODS

### Animals

Our research has been approved by our local ethics committee review board (Comité Régional d'Ethique sur l'Expérimentation Animale du Limousin, Limoges, France) and carried according the European guidelines for animal experimentation. The 3′RR deletion has been done in a 129 ES cell line and developed in a 129 background [5]. The presence of the 3′RR-deleted allele was verified by PCR. 3′RR-deficient mice (IgH a^Δ3′RR^/a^Δ3′RR^) and *wt* 129 mice (IgH a^*wt*^/a^*wt*^) were investigated. Heterozygous IgH a^Δ3′RR^/b*^wt^* mice were generated by crossing homozygous 3′RR-deficient mice (IgH a^Δ3′RR^/a^Δ3′RR^) with C57BL/6 mice (IgH b*^wt^*/b*^wt^*) mice. Mixed Sv/129 x C57BL/6 mice (IgH a*^wt^*/b*^wt^*) were used as control mice.

### PCR

PCR experiments for detection of the *wt* 3′RR allele were carried out with specific forward 5′-CCAAAAATGGCCAGGCCTAGG-3′ and reverse 5′-GA CCCTGTCCTATGGCT GAC-3′ primers. PCR experiments for detection of the deficient 3′RR allele were carried out with specific forward 5′-TCCCTGGACAATCTGCACAT-3′ and reverse 5′-GACCCTGTCCTATGGCTGAC-3′ primers. DNA was denatured 180 sec at 95°C, and then submitted to 35 cycles consisting in 94°C / 30 sec, 60°C / 30 sec and 72°C / 60 sec. Amplification products were analysed on a 1.2% agarose gel. Expected sizes of amplified products were 250 bp and 587 bp for mutated and *wt* alleles, respectively.

### Granulomas induction

To induce inflammatory process, 12-14-weeks-old mice received a single *i.p.* injection of 1 ml of 2,6,10,14 tetrametyl-pentadecane (pristine) (95%, Sigma). After 3 weeks mice were euthanized.

### Flow cytometry analysis

Gut associated whole mesenteric tissue was obtained from each animal. A single-cell suspension was obtained after filtration through a fine nylon mesh. Cells were incubated with monoclonal antibodies for 30 min at 4°C. The following antibodies were used: IgM^a^-FITC, IgM^b^-PE and B220-BV510. Labelled cells were analyzed on a Fortessa LSR2 (Beckman Coulter).

### Isolation of adherent and non adherent granuloma cells

Animals were sacrificed, gut associated whole mesenteric tissue collected and single cell suspension obtained as described above. Samples (2×10^6^ cells) were cultured in a 6 well plate at 37°C with 5% CO_2_ for 2 hours. Total RNA was isolated using Tri-Reagent (Sigma) from both adherent and non adherent cells. Samples were stored at −20°C until used.

### Cell counts

Single cell suspension of granulomas were counted and characterised in the CELL-DYN Emerald (Abbot), a compact bench-top hematology analyzer that can be used for a three-part white cell differential analysis of human and mouse samples [37].

### Real-time PCR analysis

RNA was extracted according to the manufacturer's instruction. Complementary DNA (cDNA) was synthesized with 1 μg of total RNA using the high capacity cDNA reverse transcription kit from Applied biosystems. Real-time PCR analysis was performed using TaqMan reagents: TNF-α (Mm00443258-m1), IL-6 (Mm00446190-m1), IL-12-P40 (Mm00434189-m1), CXCL2 (Mm00436450-m1), IL-10 (Mm01288386-m1), IL-4 (Mm00445259-m1), INF-γ (Mm00801778-m1), TGF-β (Mm01178820-m1) and GAPDH (Mm99999915-g1). Experiments were performed using the Step One Plus (Applied biosystems). Amounts of various transcripts were compared to sample with the lowest level of transcripts. The relative quantification of gene expression was performed using the comparative C_T_ method (ΔΔC_T_). Experiments were made in duplicate. Mean C_T_ values were used in the ΔΔC_T_ calculation by using the “relative quantitation calculation and analysis software for Applied Biosystems real-time PCR systems”.
